# Supplementary material for: The Inducible Intein-Mediated Self-Cleaving Tag (IIST) System: A Novel Purification and Amidation System for Peptides and Proteins
Source: Molecules. 2021 Sep 30;26(19):5948. doi: 10.3390/molecules26195948 (PMC8512742; doi:10.3390/molecules26195948)
Supplement: Supplementary file 1 [file molecules-26-05948-s001.zip › molecules-1369667-supplementary.pdf]

15RPC reverse-phase chromatogram

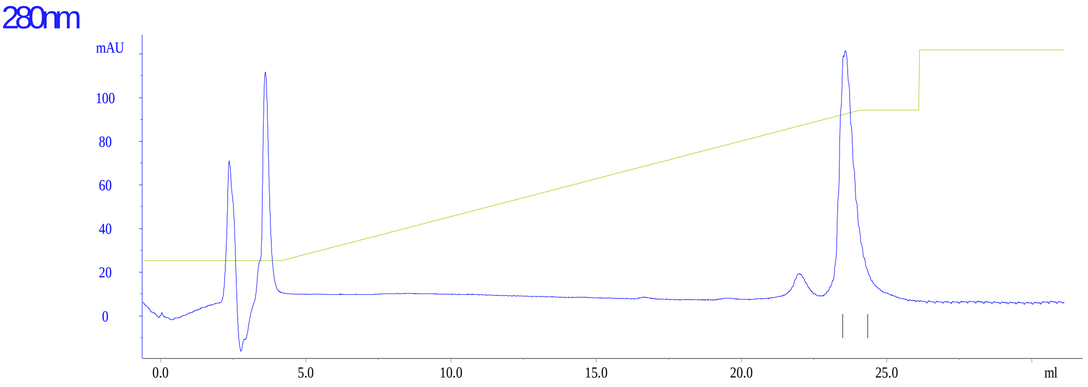

Figure S1: HPLC chromatograph of the flow-through fraction using RPC15 column. Reverse Phase Liquid Chromatography using a SOURCE 15RPC ST 4.6/100 column (GE Healthcare, USA) with a linear gradient of 30-80% (green line) from water/0.05% TFA to acetonitrile/0.05% TFA. The UV spectrum at 280 nm is shown.

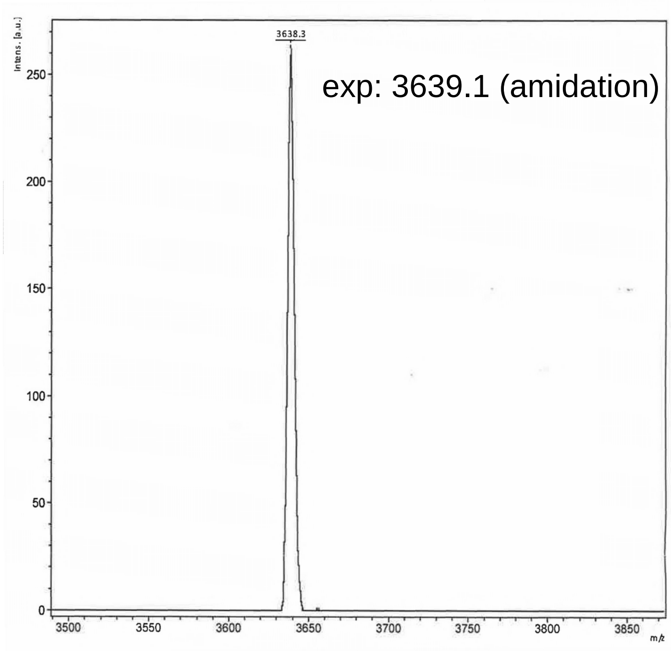

Figure S2: MALDI-TOF mass-spectrometry analysis of the major peak from HPLC (Figure S1). The expected molecular mass for the amidated GLP-1 analog is shown.
